# Experimental and Theoretical Mechanistic Study on the Thermal Decomposition of 3,3-diphenyl-4-(trichloromethyl)-5-nitropyrazoline

**DOI:** 10.3390/molecules26051364

**Published:** 2021-03-04

**Authors:** Karolina Kula, Agnieszka Kącka-Zych, Agnieszka Łapczuk-Krygier, Zbigniew Wzorek, Anna K. Nowak, Radomir Jasiński

**Affiliations:** 1 Department of Organic Chemistry and Technology, Cracow University of Technology, Warszawska 24, 31-155 Cracow, Poland; kkula@chemia.pk.edu.pl (K.K.); akacka@chemia.pk.edu.pl (A.K.-Z.); lapczuk@chemia.pk.edu.pl (A.Ł.-K.); 2 Institute of Inorganic Chemistry and Technology, Cracow University of Technology, Warszawska 24, 31-155 Cracow, Poland; wzor@indy.chemia.pk.edu.pl (Z.W.); akn@chemia.pk.edu.pl (A.K.N.)

**Keywords:** pyrazoline, nitrocompounds, thermal elimination, molecular electron density theory

## Abstract

The present paper is a continuation of comprehensive study regarding to synthesis and properties of pyrazoles and their derivatives. In its framework an experimental and theoretical studies of thermal decomposition of the 3,3-diphenyl-4-(trichloromethyl)-5-nitropyrazoline were performed. It was found, that the decompositions of the mentioned pyrazoline system in the solution and at the melted state proceed via completely different molecular mechanisms. These mechanisms have been explained in the framework of the Molecular Electron Density Theory (MEDT) with the computational level of B3LYP/6-31G(d). A Bonding Evolution Theory (BET) examination of dehydrochlorination of the 3,3-diphenyl-4-(trichloromethyl)-5-nitropyrazoline permits elucidation of the molecular mechanism. It was found, that on the contrary for most known HCl extrusion processes in solution, this reaction is realised via single-step mechanism.

## 1. Introduction

**Scheme 1:**
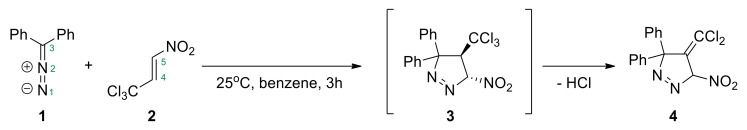
General scheme of reaction between diphenyldiazomethane (**1**) and (*E*)-3,3,3-trichloro-1-nitroprop-1-ene (**2**).

This work is a continuation of our comprehensive theoretical and experimental studies about mechanisms of elimination reactions involving different type of nitro-compounds [1,2,3,4,5,6]. Recently [1], we have established, that the [3 + 2] cycloaddition reaction between diphenyldiazomethane **1** and (*E*)-3,3,3-trichloro-1-nitroprop-1-ene **2**, can be carried out at room temperature and gives 3,3-diphenyl-4-(dichloromethylene)-5-nitropyrazoline 4 (Scheme 1) as a rare example of methylene functionalised pyrazole derivatives.

This cannot be primary reaction product because it is widely known that cycloaddition processes proceed with full atomic economy [7,8]. So, we assumed, that primary product is 3,3-diphenyl-4-(trichloromethyl)-5-nitropyrazoline **3**, which spontaneously decomposed, partially with HCl extrusion. This observation is unexpected, because the presence of CCl_3_ group generally stabilises the heterocyclic systems in the comparison with analogs with CH_3_ or other groups. This was confirmed inter alia for series of 2,3,3-triphenyl-4-nitro-5-*R*-isoxazolidines [9,10,11]. On the other hand, examples which are available in the literature, confirm relatively high energy of the activation for the dehydrochlorination reactions from CCl_3_ functionalised molecules. In the consequence, these type of reactions, require the presence of base catalysts. Antonov [12] observed that the dehydrochlorination of pyrimidine derivatives, were converted in a good yield into the corresponding 2,3-dichlorovinyl derivatives. In the same conditions (reflux and the presence of sodium ethylate) the dehydrochlorination of furopyrimidines and furylformamidines leads to mixtures of the starting material and its dichlorovinyl derivative, which could not be isolated. On the other hand, furopyrimidinonimines are readily converted to the corresponding divinyl derivatives in good yields by the action of an equivalent amount of sodium ethylate. In the case of oxofuropyrimidines the dehydrochlorination, which proceeds only partially, was not facilitate because of the lower basicity of the oxo group in comparison with the imino group [12]. The dehydrochlorination of *N*-amidoalkylated derivatives of 2-amino-1,3,4-oxadiazole in the first stage leads to *N*-(1-hetaryl-2,2-dichlorovinyl)-carboxamide and then becomes stable carboxamide [13].

Most of researches explored properties of trihalomethyl compounds. McLennan [14,15] established that the mechanism of dehydrochlorination of 1,1-diaryl-2,2,2-trichloroethanes by anionic bases in alcoholic solvents is E1cB scheme. Paciorek et al. [16] have found that the dehydrohalogenation reaction of 1,1,1-trichloroperfluoroalkanes is extremely sensitive to moisture, to the purity of the starting materials and the type of solvent. Depending on the conditions, a range of by-products was obtained. For example, the dehydrochlorination of 1,1,1-trichloro-2,2-bis(4-chlorophenyl)ethane (DDT) is catalyzed by hexadecyltrimethylammonium bromide (CTAB) micelles [17]. In case of 1,1,1-trichloro-2-arylethanes, the dehydrochlorination protocol can be based on the use of a dipolar aprotic solvent and a lithium halide under reflux conditions [18], using a methanol solution of potassium hydroxide [19], anhydrous ammonia [20] or methylamine [20].

So, in the case of the title compound, the simple thermal HCl extrusion process should be considered as forbidden from kinetic point of view. Next, known nitropyrazoline systems, decompose easily via other reaction channels such as (i) nitrogen extrusion [21,22] or (ii) nitrous acid extrusion [23]. In the case of the title reaction, these type of products from such elimination processes mentioned above, was not detected in the postreaction mixture [1].

**Scheme 2:**
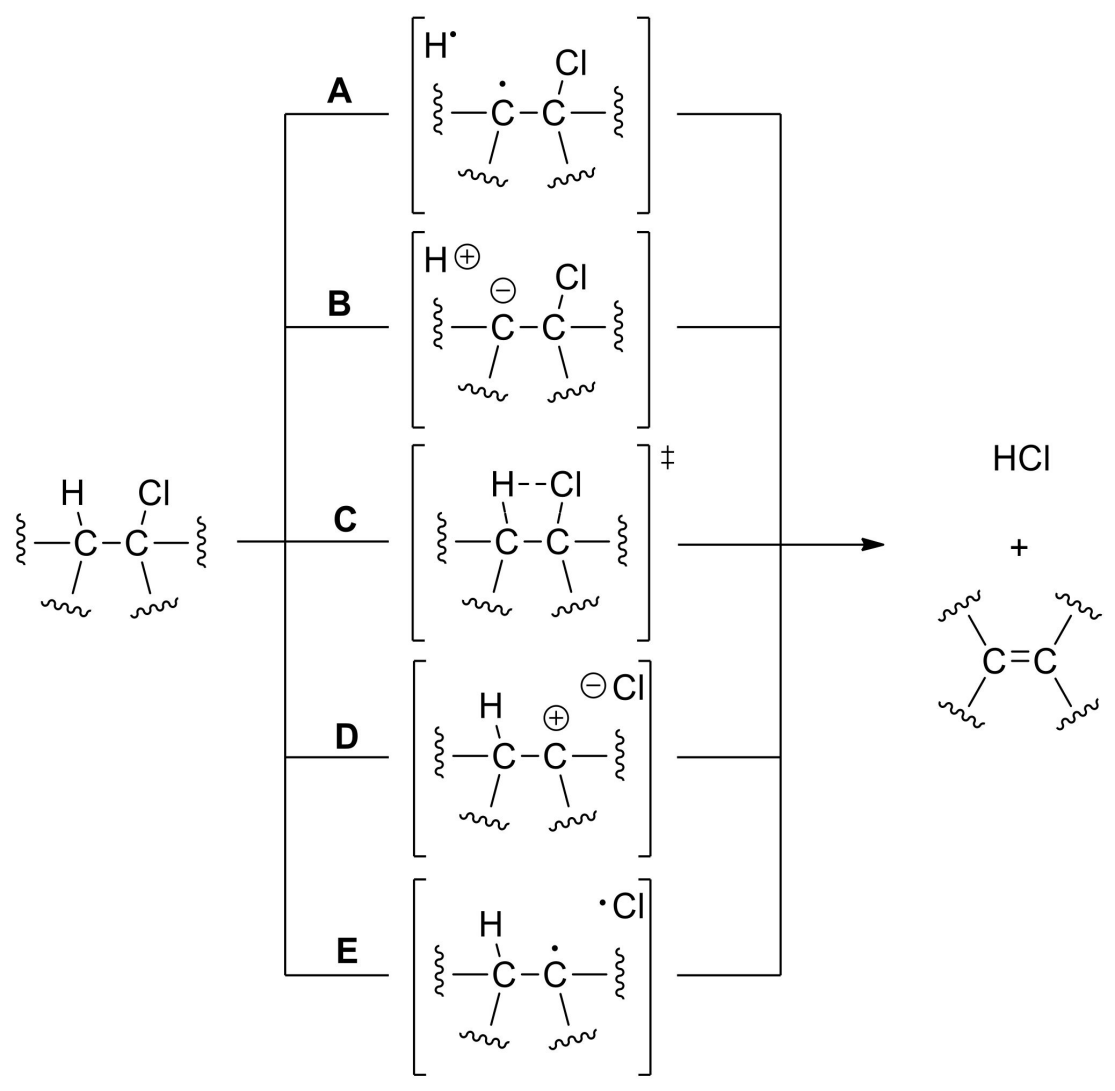
Theoretically possible mechanisms of the HCl extrusion process from organic molecules.

Additionally, it should be underlined, that different types of mechanisms can be considered regarding to the HCl elimination reactions (Scheme 2). In particular, it will be “pure” radical mechanism (**A** or **E**) [24], E1cb (**B**) or E1 (**D**) ionic mechanisms [25,26], as well as one-step mechanism (**C**) with less or higher synchronous, four-membered transition state [25,27]. Generally, for reactions in the gas phase, radical mechanisms are assumed [24], whereas for reactions in solution, ionic mechanism are considered as most probable. On the other hand, one-step mechanism **C**, is generally assumed as most less probable, due to high geometrical strains in the hypothetical transition state [27]. The question of molecular mechanism of HCl extrusion cannot be however a priori explained, without any quantum-chemical and/or laboratory experiments.

Issues mentioned above provoked us to shed a light on the molecular mechanism of decomposition of 3,3-diphenyl-4-(trichloromethyl)-5-nitropyrazoline **3**, under different conditions, and using different type of theoretical and experimental techniques. We hope, that this comprehensive study will be valuable contribution to knowing better elimination reactions in the organic chemistry.

## 2. Results and Discussion

Firstly, we decided to re-examine the [3 + 2] cycloaddition of **1** with **2** again to try to isolate the postulated primary reaction product **3**. After several experiments, we established, that in reaction proceeded at −10 °C under solvent free conditions, the isolation of individual **3** is possible. Its constitution was confirmed by spectral analysis. In particular, in the first stage we have analysed its IR spectrum. Absorption bands typical of the NO_2_ group [28] and pyrazoline ring [29] were identified in the IR spectrum. Next, on the ^1^H NMR spectrum, independently of signals of aryl rings, we detected two doublets at 5.87 ppm and 6.58 ppm, which can be interpreted as signals from two vicinal protons of pyrazoline ring (H4 and H5, respectively). This confirms the retention of the HCl moiety in the isolated compound. Next, the value of the coupling constant *J*_4,5_ (5.62 Hz) shows, that protons H4 and H5 exist on the opposite sides of the heterocyclic ring. This proves that the configuration of substituents in the cycloaddition product is the same as in the case of nitroalkene **2** and can confirm the one-step cycloaddition mechanism postulated earlier by us [1]. So, the physical analysis confirms without any doubts, that constitution of 3,3-diphenyl-4-trichloromethyl-5-nitropyrazoline should be assigned for the isolated product 3.

Next, we analysed the process of decomposition of **3** in a solution. For this purpose, we thermostated samples of **3** at 60–80 °C in different solvents such as benzene, DCM and ethanol. In all cases, we obtained dehydrochlorination product, which was identical as isolated in our work [1]. So, the thesis that **4** is produced directly via HCl extrusion from primary formed 3,3-diphenyl-4-trichloromethyl-5-nitropyrazoline **3** is fully confirmed.

Our DFT study unexpectedly detected, that this transformation is realized via single transition state. This is accompanied with the overcoming barrier of activation about 35 kcal/mol (ΔG^≠^ = 35.6 kcal/mol). This value is close to, values estimated for other one-step elimination processes, which realized under mild conditions [3]. Next, the analysed HCL elimination process should be considered as irreversible from thermodynamic point of view (ΔG = −13.6 kcal/mol).

**Figure 1:**
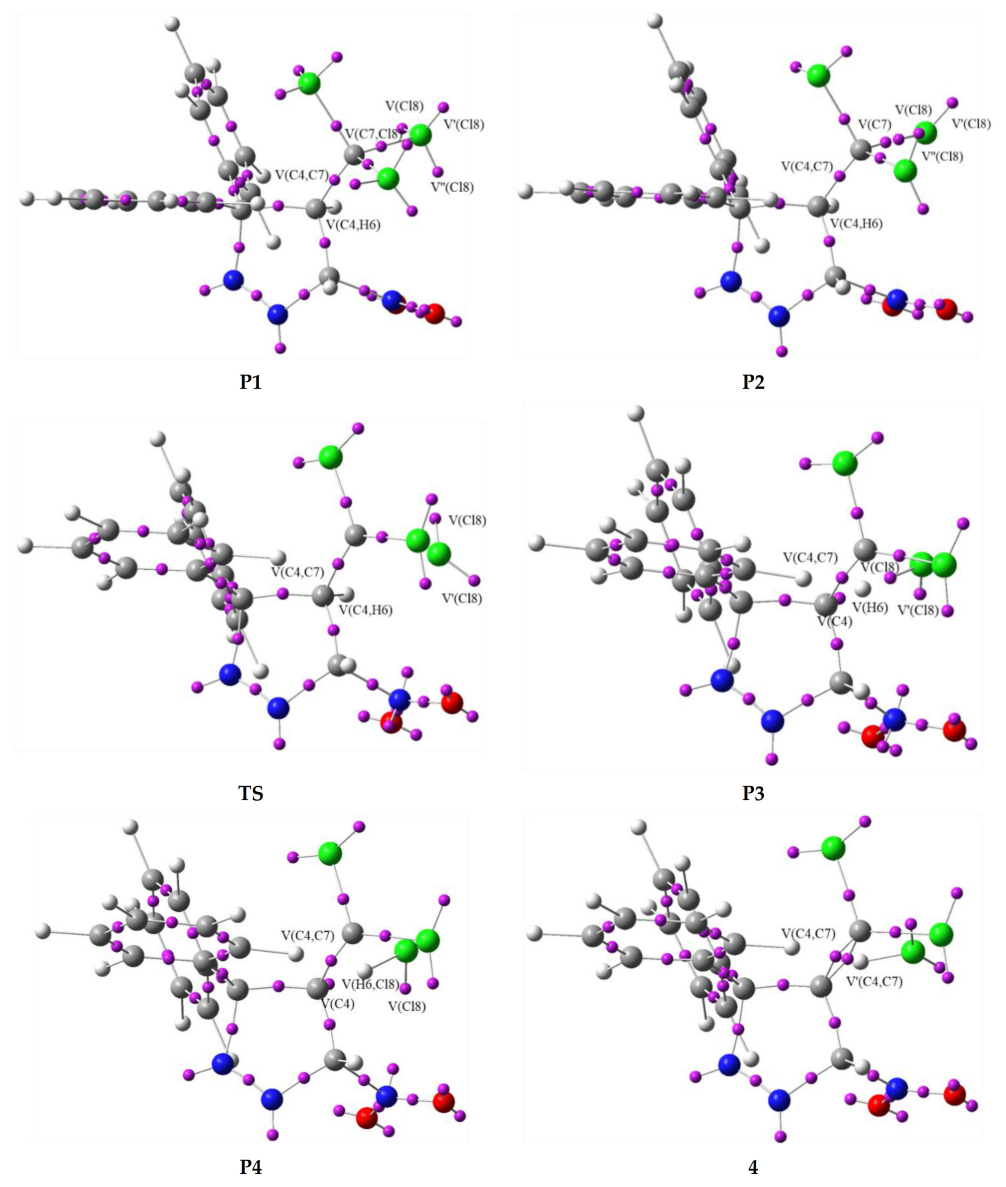
ELF atracttor positions for the points of the IRC defining Phases I-VI along the dehydrochlorination of **3** into **4**.

In order to understand the bonding changes along the **3**→**4** transformation, a BET [30] analysis was performed. BET study of this transformation, indicates that this reaction is topologically characterized by six different phases. The population of the most significant valence basins of the selected points of the IRC, Pi, defining the different topological phases, is included in Table 1. The attractor positions of the ELF for the relevant points along the IRC are shown in Figure 1.

*Phase I*, 2.13 Å ≤ d(C4-H6) < 2.15 Å, 2.95 Å ≥ d(C4-C7) > 2.93 Å, 3.45 Å ≤ d(C7-Cl8) < 3.89 Å, 4.65 Å ≥ d(H6-Cl8) > 4.56 Å, begins at **P1**, being a first structure of the reaction path between **3** and **TS**. The ELF picture of **P1** exhibits the topological characteristics of the molecules **3**. In this phase, only small changes in the population of the valence basins of **P1** compared to **3** are observed. The population of V(C4,C7) and V(C7,Cl8) disynaptic basin progressively increases as well the population of V(C7,Cl8) disynaptic basin progressively decreased (Table 1).

*Phase II*, 2.15 Å ≤ d(C4-H6) < 2.17 Å, 2.93 Å ≥ d(C4-C7) > 2.77 Å, 3.89 Å ≤ d(C7-Cl8) < 5.79 Å, 4.56 Å ≥ d(H6-Cl8) > 4.15 Å, starts at **P2**. The first noticeable topological change along the IRC path occurs in this phase; the V(C7,Cl8) dinsyptic basin disappears; and a new V(C7) monosynaptic basin, integrating 0.78 e, is created at **P2**. At this point, we also observed the increases the total population of V(Cl), V′(Cl) and V″(Cl) monosynaptic basins. The electron-density of this basin mainly proceeds from the depopulation of the C7-Cl8 single bond.

*Phase III*, 2.17 Å ≤ d(C4-H6) < 2.78 Å, 2.77 Å ≥ d(C4-C7) > 2.65 Å, 5.79 Å ≤ d(C7-Cl8) < 6.05 Å, 4.15 Å ≥ d(H6-Cl8) > 3.13 Å, begins at **TS**. In this phase, the next topological change along the reaction path take place; the V(C7) monosynaptic basin disappears and the population of V(C4,C7) increases. The value of the V(C4,H6) disynaptic basin reaches the minimum in this phase and the three V(Cl8), V′(Cl8) and V″(Cl8) monosynaptic basins, present at **P2** have merged into two V(Cl8) and V′(Cl8) monosynaptic basins integrating 7.58 e. The transition state **TS** of this reaction is found in this phase, d(C4-H6) = 2.17 Å, d(C4-C7) = 2.77 Å, d(C7-Cl8) = 5.79 Å and d(H6-Cl8) = 4.15 Å (Figure 1). These changes are related to a high energy cost of 36.9 kcal/mol (Table 1).

At *Phase IV*, 2.78 Å ≤ d(C4-H6) < 2.99 Å, 2.65 Å ≥ d(C4-C7) > 2.63 Å, 6.05 Å ≥ d(C7-Cl8) > 6.04 Å, 3.13 Å ≥ d(H6-Cl8) > 2.91 Å, which starts at **P3**, the next most significant topological change along the reaction path take place. The V(C4,H6) disynaptic basin disappears and a two new V(C4) and V(H6) monosynaptic basins are created, integrating 0.86 e and 0.79 e, respectively. This topological change is related with the rupture of the single C4-H6 bond.

*Phase V*, 2.99 Å ≤ d(C4-H6) < 3.47 Å, 2.63 Å ≥ d(C4-C7) > 2.58 Å, 6.04 Å ≥ d(C7-Cl8) > 6.01 Å, 2.91 Å ≥ d(H6-Cl8) > 2.55 Å, starts at **P4**. At this point, we observed the disappearance of V(H6) monosynaptic basin; the two V(Cl8) and V′(Cl8) monosynaptic basins, present at **P5** have merged into one V(Cl8) monosynaptic basin, with decrease the population to 6.85 e; and the formation of a new V(H6,Cl8) disynaptic basin, integrating 1.33 e (Figure 1).

Finally, the last Phase VI, 3.47 Å ≤ d(C4-H6) < 6.58 Å, 2.58 Å ≥ d(C4-C7) > 2.53 Å, 6.01 Å ≤ d(C7-Cl8) < 6.48 Å, 2.55 Å ≥ d(H6-Cl8) > 2.50 Å, is located between points **P5** and **4**. At this point, the last relevant change along the reaction path take place; the V(C4,C7) disynaptic basin splits into two new V(C4,C7) and V′(C4,C7) disynaptic basins integrating 2.05 e and 1.95 e, respectively. This change is related with the formation of a double bond between C4-C7 atoms in molecule **4**. The energy of the reaction is −2.3 kcal/mol.

**Figure 2:**
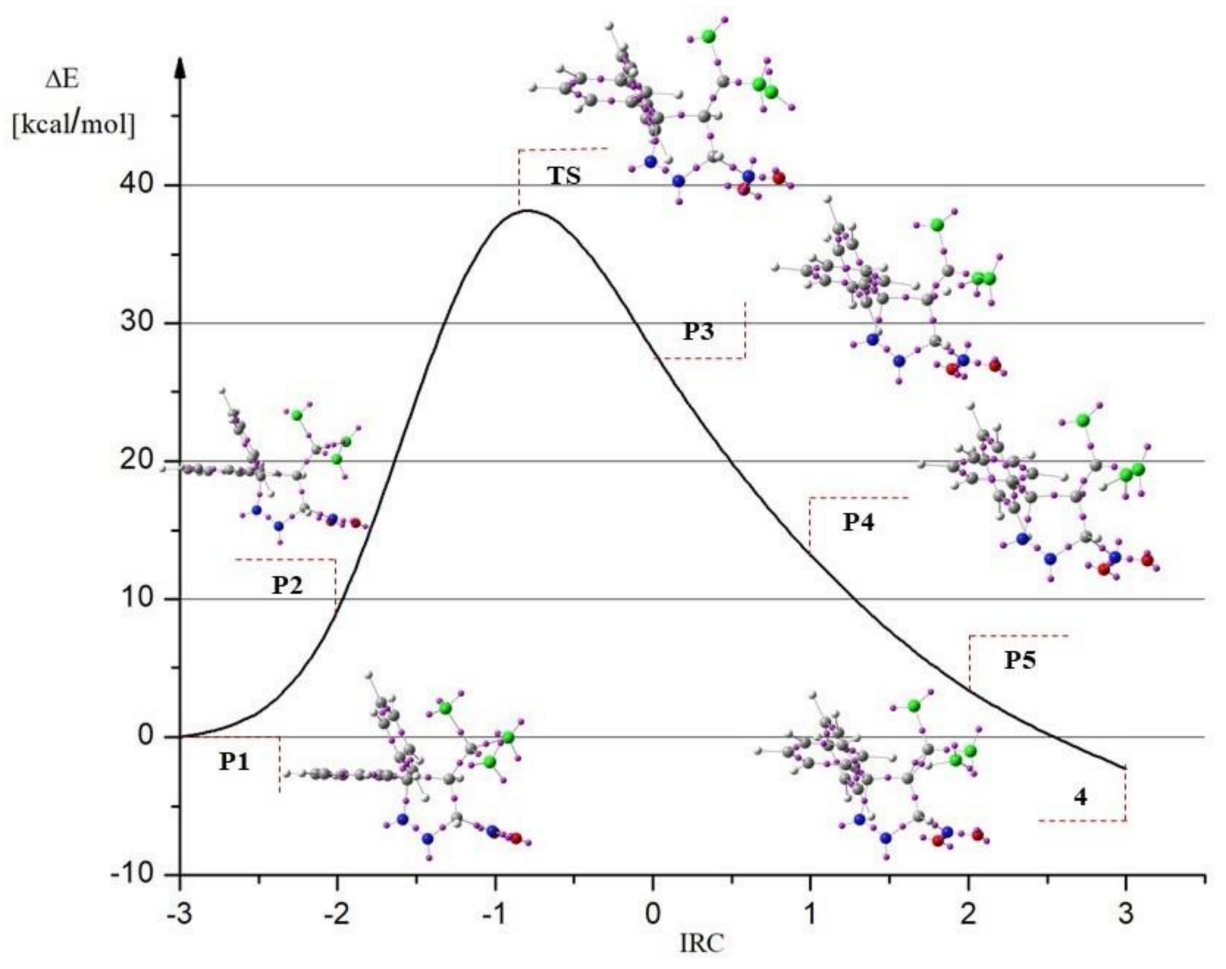
B3LYP(PCM)/6-31G(d) IRC profile including the ELF attractor positions for the most relevant points of the dehydrochlorination of **3** and relative electronic energies.

Some appealing conclusion can be drawn from this BET analysis: (i) the molecular mechanism of dehydrochlorination of **3** can be topologically characterized by six different phases, which have been grouped into four groups A-D and linked to significant chemical events (Table 2, Figure 2); (ii) Group A, containing *Phases I* and *II*, is associated with the rupture of the C7-Cl8 single bond and formation of a V(C7) monosynaptic basin integrating at basin population 0.78 e, which can be associated with formation of C7 pseudoradical centre; (iii) Group B, comprises *Phases III* and *IV*, in which we observed the breaking the C4-H6 single bond, formation of new C4 pseudoradical centre and V(H6) monosynaptic basin and disappearance of C7 pseudoradical centre; (iv) Group C, containing *Phase V* is mainly associated with formation of H6-Cl8 single bond and disappearance of V(H6) monosynaptic basin; (v) last Group D, containing *Phase VI* in which we observed the disappearance of C7 pseudoradical centre and formation of C4=C7 double bond in molecule **4**. It should also be mentioned that DFT study using different MPWB1K functional with 6-311G(d,p) basic set gives a similar representation of this reaction. In the case of this calculations based on MPWB1K(PCM) functional, we observed increase in the relative energy for all of the reaction points along the IRC path. MPWB1K(PCM)/6-311G(d,p) the population of the most significant valence basins of the selected points of the IRC, Pi, defining the different topological phases, is included in Table S1 of the Supplementary Information’s.

**Figure 3:**
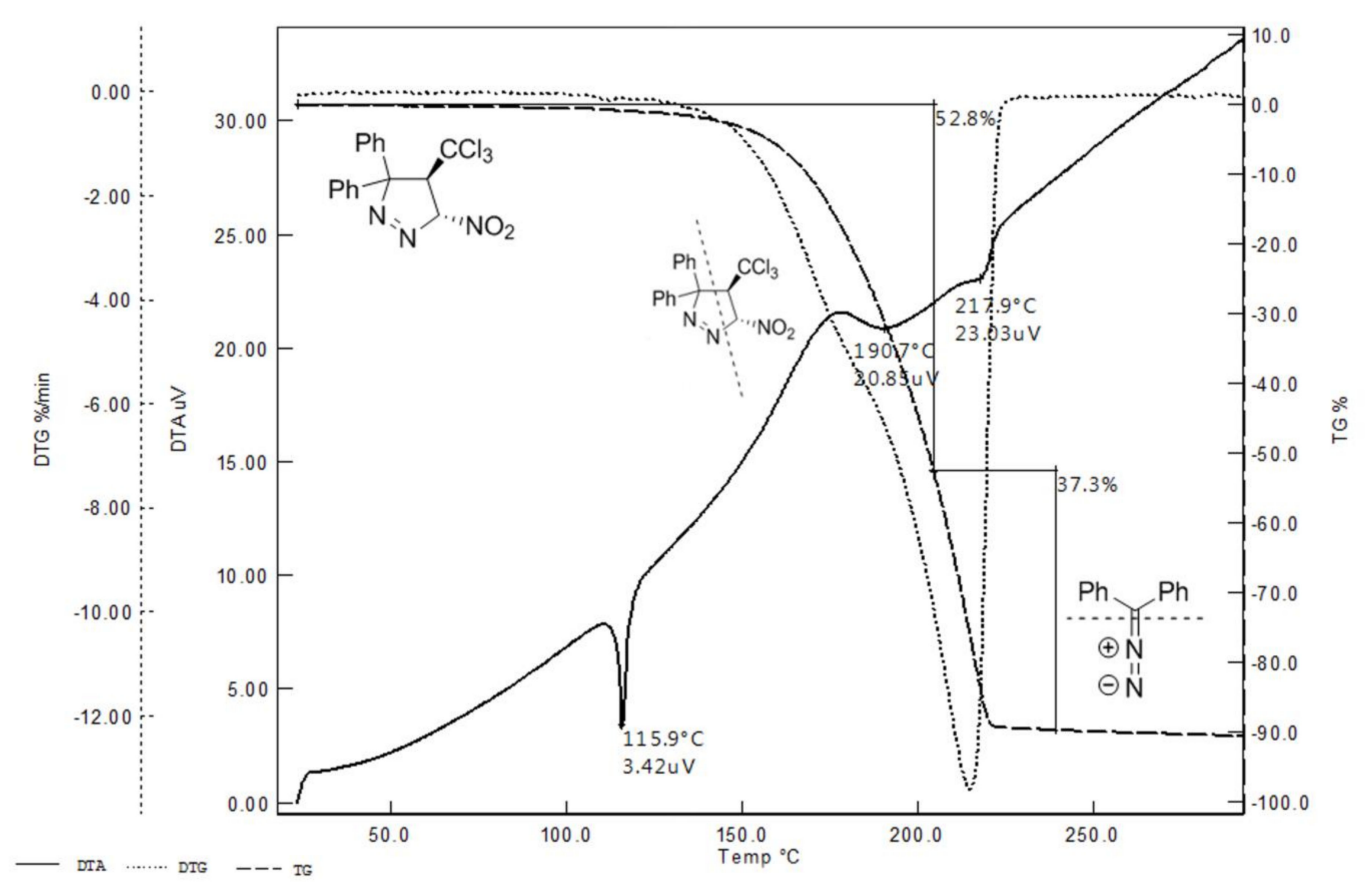
The course of decomposition of 3,3-diphenyl-4-(trichloromethyl)-5-nitropyrazoline **3** during heating in the range of 20–300 °C.

During the structural analysis of pyrazoline **3**, we established, that this compound melts at about 116 °C without the decomposition. This suggests, that molecular mechanism of the decomposition of **3** without presence of the solvent should be substantially different as the one in the solution. To support this thesis, we performed comprehensive thermal analysis of this transformation. It was found that the decomposition of 3,3-diphenyl-4-(trichloromethyl)-5-nitropyrazoline **3** takes place in the melted state. Its melting temperature is 115.9 °C. In the first step at temperature 190.7 °C, 3,3-diphenyl-4-(trichloromethyl)-5-nitropyrazoline **3** breaks down into two separate molecules (Figure 3). The first molecule, (*E*)-3,3,3-trichloro-1-nitroprop-1-ene **2**, goes into the gaseous phase. The decrease in the value of sample mass observed in the diagram was 52.8% and is close to the theoretical 52.9%. The second molecule, diphenyldiazomethane **1**, decomposes at 217.9 °C, giving off a nitrogen molecule. The total weight loss during heating was 90.1%, which indicates that diphenyldiazomethane **1** decomposition is accompanied by diphenylcarbene decomposition, forming a char at the bottom of the crucible.

Thus, the thermal analysis of the considered decomposition processes suggest, that they proceed according to the retro-[3 + 2] cycloaddition scheme. Analogous process in the benzene solution has recently been examined by us in the detail [1]. This study confirmed hypothesis mentioned above. Our actual quantum-chemical calculations show, that retro-[3 + 2] cycloaddition involving 3,3-diphenyl-4-(trichloromethyl)-5-nitropyrazoline **3** without the presence of the solvent exhibits similar nature. In particular, a BET analysis allows to emphasis ten different topologically phases associated with mentioned retro-[3 + 2] cycloaddition. In the first stage of the process, we observed the rupture of the N1-C5 single bond and formation of C5 *pseudoradical* centre and N1 lone pairs. The next step in the reaction is rupture of the second C3-C4 single bond and formation of two C3 and C4 *pseudoradical* centres. In the last stage of the reaction we noticed the formation of two N1=N2 and C4=C5 double bonds.

## 3. Materials and Methods

### 3.1. Instrumentation

In the course of our experiments, equipments located within Department of Chemical Engineering and Technology of Cracow University of Technology were applied. Melting points were determined on a Boetius apparatus and are uncorrected. HR-MS spectra were performed on a Shimadzu LCMS-IT-TOF instrument with ES ionization (heat bock and CDL temperature 200 °C, nebulising gas flow 1.5 cm^3^/min), connected to Shimadzu Prominence chromatograph two pumps LC-20AD equipped with PhenomenexKinetex 2.6 µm C18 100A column (65% acetonitrile was used as the eluent). IR spectra were recorded in Thermo Fisher Scientific Nicolet IS 10 FT-IR using KBr pellets. Omnic software was used to visualize the obtained IR spectra. ^1^H NMR (500 MHz) and ^13^C NMR (125 MHz) spectra were recorded in a Bruker AMX 500 spectrometer. Chemical shifts (*δ*) are expressed on parts per million (ppm) relative to external reference TMS. Coupling constants (*J*) are given in Hertz. The NMR spectra were performed in CDCl_3_ and referenced to the residual peak of CHCl_3_ at *δ*H = 7.26 ppm for 1H. ACD/NMR predictor was used to visualize the obtained NMR spectra. High-pressure liquid chromatography (HPLC) was done using a Knauer apparatus equipped with a UV-VIS detector. To monitor the reaction progress, a LiChrospher 18-RP 5μm column (4 × 240 mm) and 75% methanol as the eluent at the flow rate of 1.5 cm^3^/min were used. Thermal analysis of the material was performed with the use of EXSTAR SII TG/DTA 7300 apparatus. The tests were carried out in a platinum crucible, in the temperature range of 20–300 °C, in an air atmosphere with a flow of 100 cm^3^/min.

### 3.2. Materials

Commercially available (Sigma-Aldrich Poland, Szelągowska 30, 61-626 Poznań) reagents and solvents were used. All solvents have been tested by means of gas chromatography immediately before were used. Diphenyldiazomethane **1** and (*E*)-3,3,3-trichloro-1-nitro-prop-1-ene **2** were prepared in multistep reactions described earlier in literature [31,32].

#### 3.2.1. Synthesis of 3,3-Diphenyl-4-(trichloromethyl)-5-nitropyrazoline **3**

A mixture of 20 mmol of diphenyldiazomethane **1** (Equation(1)) and 30 mmol of (*E*)-3,3,3-trichloro-1-nitroprop-1-ene **2** (1.5 eq.) was stirred in the dark at −10 °C for 3 h. The excess of the nitroalkene was evaporated in vacuum. The obtained product was washed first with petroleum ether and then cold ethanol. Its physical characteristic is listed below:

3,3-Diphenyl-4-(trichloromethyl)-5-nitropyrazoline **3**: White crystals; yield 95%; m.p. 116 °C. UV/VIS spectrum λ_max_ nm.: 204; IR spectrum (KBr) cm^–1^: 1558 (–N=N–), 1495 (–NO_2_), 1359 (–NO_2_); ^1^H NMR spectrum, δ, ppm: 7.97 (2H, d, *J* = 7.3 Hz, H_Ar_), 7.54 (2H, t, *J* = 7.3 Hz, H_Ar_), 7.47–7.44 (1H, m, H_Ar_), 7.35–7.30 (3H, m, H_Ar_), 6.81 (2H, d, *J* = 8.2 Hz, H_Ar_), 6.58 (1H, d, *J* = 6.6 Hz, >CH–NO_2_), 5.87 (1H, d, *J* = 6.6 Hz, >CH–CCl_3_). ^13^C NMR spectrum, δ, ppm: 140.0; 129.3; 128.8; 127.5; 106.6; 87.6; 62.1; 40.9.

#### 3.2.2. Dehydrochlorination of 3,3-Diphenyl-4-(trichloromethyl)-5-nitropyrazoline **3** –General Procedure

A solution of 10 mmol of 3,3-diphenyl-4-(trichloromethyl)-5-nitropyrazoline **3** in 5 cm^3^ of dry solvent (benzene, DCM, ethanol) was thermostated at 60–80 °C for 3 h. The reaction progress was monitored by HPLC. The postreaction mixture was evaporated to dryness, and recrystallized from ethanol. On this way, 3,3-diphenyl-4-(dichloromethylene)-5-nitropyrazoline **4** with practical quantitative yield was obtained. Its physical characteristics is identical, as described by us earlier [1].

### 3.3. DFT Computational Study

All computations associated with the decomposition of 3,3-diphenyl-4-(trichloromethyl)-5-nitropyrazoline **3** were performed using the GAUSSIAN 16 package [33] in the Prometheus computer cluster of the CYFRONET regional computer center in Cracow. The geometries of all substrates, transition state structures (TSs) and products of the reactions were fully optimized using the B3LYP [34,35] and MPWB1K [36] functionals together with the 6-31G(d) and 6-311G(d,p) basis sets. Then, Gibbs free energies for optimized critical structures were computed using data of vibrational analysis. Stationary points were checked by vibrational frequency analyses to see whether they constituted minima or maxima on the potential energy surface (PES). All transition structures showed a single imaginary frequency (νi), whereas reactant and products had none. The polarity of the reaction environment was simulated using a relatively simple self-consistent reaction field (SCRF) [37,38,39] based on the polarizable continuum model (PCM) of Tomasi’s group [40,41]. A close enough approach to analyzing other many different reactions have been used successfully in many other works [4,42,43,44,45,46]. The topological analyses of the electron localization function (ELF) [47,48,49] were performed with the TopMod [50]. For the BET [30] studies, the topological analysis of the ELF along the IRC was performed for a total of 198 nuclear configurations for reaction of the dehydrochlorination of **3**.

## 4. Conclusions

In this paper, we have conducted an experimental and theoretical study of the thermal decomposition of the 3,3-diphenyl-4-(trichloromethyl)-5-nitropyrazoline **3**. Decomposition of **3** in solution proceeds unexpectedly with formation of **4** via HCl extrusion stage. The molecular mechanism of this process was investigated and explained based on the MEDT. The BET analysis allowed to distinguish six different phases associated with dehydrochlorination of **3**. The reaction starts with rupture of the two C7-Cl8 and C4-H6 single bonds and formation of two C7 and C4 pseudoradical centers and Cl8 and H6 lone pairs. In the next stage, we observed formation of H6-Cl8 single bond and the last stage is related to formation of C4=C7 double bond. In the contrary, the decomposition of 3,3-diphenyl-4-(trichloromethyl)-5-nitropyrazoline **3** in the melted state proceed according to the different-type mechanism. Thermal analysis as well as MEDT computational study shows, that this transformation should be considered as single-step retro-[3 + 2] cycloaddition process. The BET analysis of the retro-[3 + 2] cycloaddition allows to highlight ten different topologically phases associated with rupture and formation bonds. At the beginning of the reaction, we observed the rupture of the N1-C5 and C3-C4 single bonds and formation of C5, C3 and C4 pseudoradical centers. In turn, the formation of N1=N2 and C4=C5 double bonds takes place in subsequent stages.

## Figures and Tables

**Table 1:** B3LYP(PCM)/6-31G(d) ELF valence basin populations, distances of the breaking and forming bonds and relative^a^ electronic energies of the IRC points, **P1–4**, defining the sixdifferent phases characterising the reaction of the dehydrochlorination of **3**. The stationary points **3**, **TS** and **4** are also included. Distances are given in angstroms, Å, electron populations in average number of electrons, e, relative energies in kcal·mol^−1^.

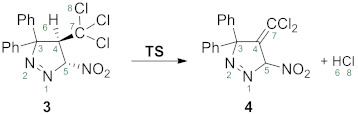														
Structures	3	P1		P2		TS		P3		P4		P5		4
Phases			*I*		*II*		*III*		*IV*		*V*		*VI*	
d(C4-H6)	2.126	2.125		2.145		2.169		2.777		2.994		3.472		6.576
d(C4-C7)	2.919	2.951		2.931		2.771		2.649		2.633		2.576		2.525
d(C7-Cl8)	3.445	3.449		3.889		5.791		6.051		6.040		6.012		6.483
d(H6-Cl8)	5.232	4.649		4.562		4.150		3.128		2.914		2.551		2.503
ΔE ^a^		0.0		9.2		36.9		28.0		13.2		3.4		−2.3
V(C4,H6)	2.03	2.02		2.01		1.95								
V(C4)								0.86		0.79				
V(H6)								0.79						
V(C4,C7)	2.07	2.09		2.10		2.42		2.95		3.12		2.05		2.00
V′(C4,C7)												1.95		1.99
V(C7)				0.78										
V(C7,Cl8)	1.44	1.48												
V(Cl8)	2.36	2.51		3.05		3.75		6.99		6.85		6.39		6.28
V′(Cl8)	1.89	1.83		1.95		3.83		0.46						
V″(Cl8)	2.21	2.10		1.98										
V(H6,Cl8)										1.33		1.68		1.69

^a^ Relative to the first point of the IRC, **P1**.

**Table 2:** Sequential bonding changes along the reaction of dehydrochlorination of **3** leading to formation of **4**, showing the equivalence between the topological characterisation of the different phases and the chemical processes occurring along them. Distances are given in angstroms, Å and relative energies referred to the first point on IRC path (**P1**) are given in kcal/mol.

Group	Phases	d_1_(C4-H6) d_2_(C4-C7) d_3_(C7-Cl8) d_4_(H6-Cl8)	∆E	Topological Characterization	Chemical Process
*A*	*I-II*	2.13 ≤ d_1_ < 2.15 2.95 ≥ d_2_ > 2.93 3.45 ≤ d_3_ < 3.89 4.65 ≥ d_4_ > 4.56	9.2	Disappearance of V(C7,Cl8) disynaptic basin and formation of V (C7) monosynaptic basin	Rupture of the C7-Cl8 bond and formation of C7 *pseudoradical* centre
*B*	*III-IV*	2.15 ≤ d_1_ < 2.99 2.93 ≥ d_2_ > 2.63 3.89 ≤ d_3_ < 6.04 4.56 ≥ d_4_ > 2.91	28.0	Disappearance of V(C4,H6) disynaptic and V(C7) monosynaptic basins and formation of V(C4) and V(H6) monosynaptic basins	Rupture of the C4-H6 bond and formation of C4 *pseudoradical* centre
*C*	*V*	2.99 ≤ d_1_ < 3.47 2.63 ≥ d_2_ > 2.58 6.04 ≤ d_3_ < 6.01 2.91 ≥ d_4_ > 2.55	13.2	Formation of V(H6,Cl8) disynaptic basin and disappearance of V(H6) monosynaptic basin	Formation of the H6-Cl8 bond
*D*	*VI*	3.47 ≤ d_1_ < 6.58 2.58 ≥ d_2_ > 2.53 6.01 ≤ d_3_ < 6.48 2.55 ≥ d_4_ > 2.50	−2.3	Formation of V(C4,C7) disynaptic basin and disappearance of V(C4) monosynaptic basin	Formation of the C4=C7 double bond

## Data Availability

The data presented in this study are available on request from the corresponding author.

## Supplementary Materials

Table S1: MPWB1K(PCM)/6-311G(d,p) ELF valence basin populations, distances of the breaking and forming bonds and relative^a^ electronic energies of the IRC points, P1 – 4, defining the six different phases characterizing the reaction of the dehydrochlorination of **3**. The stationary points **3**, **TS** and **4** are also included. Distances are given in angstroms, Å, electron populations in average number of electrons, e, relative energies in kcal·mol^−1^.

## Sample Availability

Samples of the compounds are available from the corresponding authors.
