# Supplementary material for: Experimental and Theoretical Mechanistic Study on the Thermal Decomposition of 3,3-diphenyl-4-(trichloromethyl)-5-nitropyrazoline
Source: Molecules. 2021 Mar 4;26(5):1364. doi: 10.3390/molecules26051364 (PMC7961919; doi:10.3390/molecules26051364)
Supplement: Supplementary file 1 [file molecules-26-01364-s001.pdf]

Supporting Information

Experimental and Theoretical Mechanistic Study on the Thermal Decomposition of 3,3-diphenyl-4-(trichloromethyl)-5-nitropyrazoline

Karolina Kula [1], Agnieszka Kącka-Zych [1], Agnieszka Łapczuk-Krygier [1], Zbigniew Wzorek [2], Anna Nowak [2] and Radomir Jasiński [1,*]

[1] Department of Organic Chemistry and Technology, Cracow University of Technology, Warszawska 24, 31-155 Cracow, Poland
[2] Institute of Inorganic Chemistry and Technology, Cracow University of Technology, Warszawska 24, 31-155 Cracow, Poland

Table S1. MPWB1K(PCM)/6-311G(d,p) ELF valence basin populations, distances of the breaking and forming bonds and relative[a] electronic energies of the IRC points, P1 – 4, defining the six different phases characterizing the reaction of the dehydrochlorination of **3**. The stationary points **3**, **TS** and **4** are also included. Distances are given in angstroms, Å, electron populations in average number of electrons, e, relative energies in kcal·mol^{-1}.

Structures	**3**	**P1**	**P2**	**TS**	**P3**	**P4**	**P5**	**4**
Phases			*I*	*II*	*III*	*IV*	*V*	*VI*
d(C4-H6)	2.128	2.127	2.143	2.170	2.787	2.991	3.412	6.706
d(C4-C7)	2.929	2.931	2.899	2.781	2.657	2.642	2.563	2.515
d(C7-Cl8)	3.455	3.459	3.903	5.801	6.049	6.029	6.013	6.449
d(H6-Cl8)	5.248	4.689	4.564	4.160	3.114	2.923	2.641	2.523
ΔE[a]		0.0	12.3	41.9	35.7	18.5	5.6	2.4
V(C4,H6)	2.02	2.02	2.01	1.95				
V(C4)					0.86	0.75		
V(H6)					0.77			
V(C4,C7)	2.08	2.09	2.10	2.33	2.86	3.05	1.95	2.05
V′(C4,C7)							1.93	1.90
V(C7)			0.79					
V(C7,Cl8)	1.54	1.56						
V(Cl8)	2.35	2.36	2.81	4.04	6.93	6.74	6.43	6.34
V′(Cl8)	1.91	1.90	1.89	3.65	0.55			
V′′(Cl8)	2.10	2.12	1.98					
V(H6,Cl8)						1.34	1.63	1.64

[a]Relative to the first point of the IRC, **P1**.
